# DMM Outstanding Paper Prize 2024 winners: Destynie Medeiros, Karen Ayala Baylon, Hailey Egido-Betancourt, Christopher Chapleau and Wei Li, and Jasmin Scheurer and Birgit Sauer

**DOI:** 10.1242/dmm.052481

**Published:** 2025-06-02

**Authors:** Rachel Hackett

**Affiliations:** The Company of Biologists, Bidder Building, Station Road, Cambridge CB24 9LF, UK

## Abstract

Disease Models & Mechanisms (DMM) is delighted to announce that the winners of the DMM Outstanding Paper Prize 2024 are Destynie Medeiros, Karen Ayala Baylon, Hailey Egido-Betancourt, Christopher Chapleau and Wei Li for their Research Article (titled ‘A small-molecule TrkB ligand improves dendritic spine phenotypes and atypical behaviours in female Rett syndrome mice’), and Jasmin Scheurer and Birgit Sauer for their Resources & Methods article (titled ‘Histological and functional characterization of 3D human skin models mimicking the inflammatory skin diseases psoriasis and atopic dermatitis’). The two prizes of £1000 are awarded to the first author(s) of the papers that are judged by the journal's Editors to be the most outstanding contribution to the journal that year.

## Teaser

**Summary:** DMM announces the winners of the DMM Outstanding Paper Prize for Research and Resource articles published in 2024.

## Outstanding Paper Prize winner for Research Articles: Destynie Medeiros, Karen Ayala-Baylon, Hailey Egido-Betancourt, Christopher Chapleau and Wei Li

### Destynie Medeiros


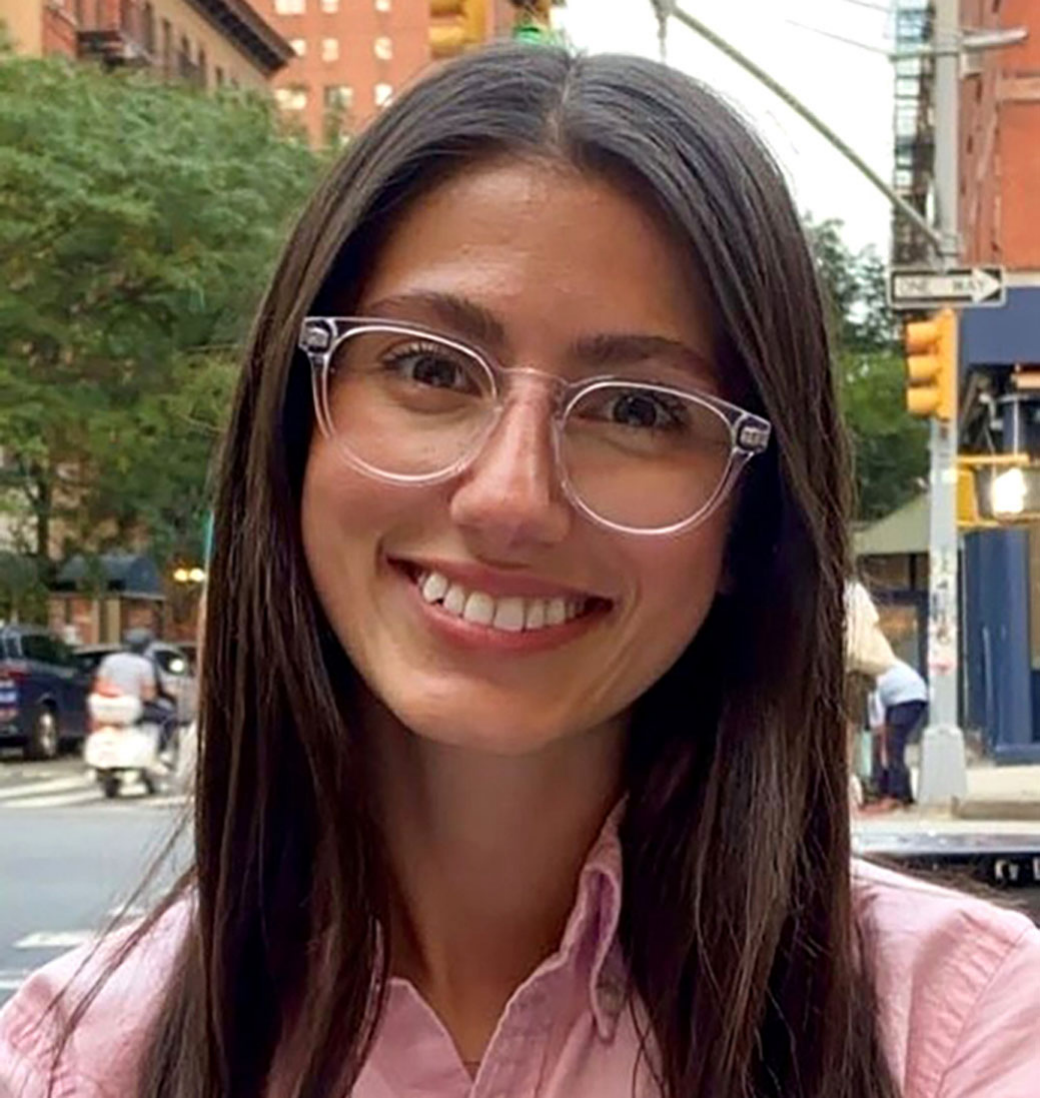


**Destynie Medeiros**

Destynie is a first-generation neuroscientist with expertise in social behaviours, dendritic spine phenotypes and *in vivo* Ca^2+^ imaging. She started her scientific journey at the University of Hartford (CT, USA) in 2015, where she studied biology and conducted her undergraduate honors research in Paola Sacchetti's lab. Destynie's research investigated the therapeutic targeting of the gut−brain axis in Alzheimer's disease progression, which was supported by the Dorothy Goodwin Scholars Program and the Undergraduate Student Government Association. This research resulted in multiple poster and oral presentations at various levels, culminating in a co-first-author manuscript. Similarly, Destynie's interests in immune interactions on neural function allowed her to explore the bi-directional connection between immunity and circadian rhythm as a co-first-author on a review article. Her dedication to a novel research project, her academic accomplishments and community outreach, led to her recognition through the Elisabeth Swain Biology Award for Academic Achievement in 2018 and being awarded the Belle K. Ribicoff Prize at her undergraduate commencement.

These experiences led Destynie to pursue a career in neuroscience and join the Neuroscience program at the University of Alabama at Birmingham (UAB), AL, USA in 2019, where she joined Lucas Pozzo-Miller's lab as PhD student. Her doctoral research, conducted in close collaboration with a dedicated team of colleagues, focused on uncovering deficits in social behaviour, and the underlying cellular and synaptic alterations in mouse models of Rett syndrome (RTT), a neurodevelopmental disorder primarily caused by pathological variants in the X-linked *MECP2* gene. As *Mecp2* regulates the expression of brain-derived neurotrophic factor (BDNF) and BDNF levels are reduced in *Mecp2-*deficient mice, the team aimed to therapeutically target this pathway. However, due to BDNF's low blood-brain barrier permeability, the group tested LM22A-4, a synthetic brain-penetrant small-molecule ligand of TrkB receptors, on dendritic spine density and behavioural phenotypes in female *Mecp2*-deficient mice, a mouse model of RTT. They found that systemic LM22A-4 treatment improved dendritic spine abnormalities and aggression-like social behaviour phenotypes. This collaborative work led to Destynie's first-author publication in Disease Models & Mechanisms (DMM), highlighting LM22A-4's potential to address dendritic spine dysgenesis and atypical behaviours in RTT. This project was deeply meaningful to Destynie's scientific and professional development, made possible by the dedication and collaboration of so many individuals who shared a passion for discovery and a commitment to supporting young scientists. It had grown over several generations of researchers in Lucas Pozzo-Miller's lab and thrived because of his unwavering mentorship, encouragement and genuine enthusiasm for both the science as well as the people behind it.

Destynie's dissertation research also explored the impairment of social memory in *Mecp2*-deficient male mice, identifying disruptions in the monosynaptic projection from the ventral hippocampus to the medial prefrontal cortex (mPFC). Collaborating with Wei Li, she developed a novel 4-chamber social arena and used *in vivo* Ca^2+^ imaging with fibre photometry to characterize mPFC pyramidal neuron and parvalbumin-expressing interneuron activity during social behaviour. Destynie and her colleagues discovered cell-type asynchrony underlying social deficits, leading to another manuscript currently in preparation of which she is also the first author.

In 2024, Destynie joined the lab of Denise Cai at the Icahn School of Medicine at Mount Sinai (NY, USA) as a postdoctoral fellow, where she is expanding her expertise of *in vivo* Ca^2+^ imaging and memory integration across hippocampal circuits.

### Karen Ayala-Baylon


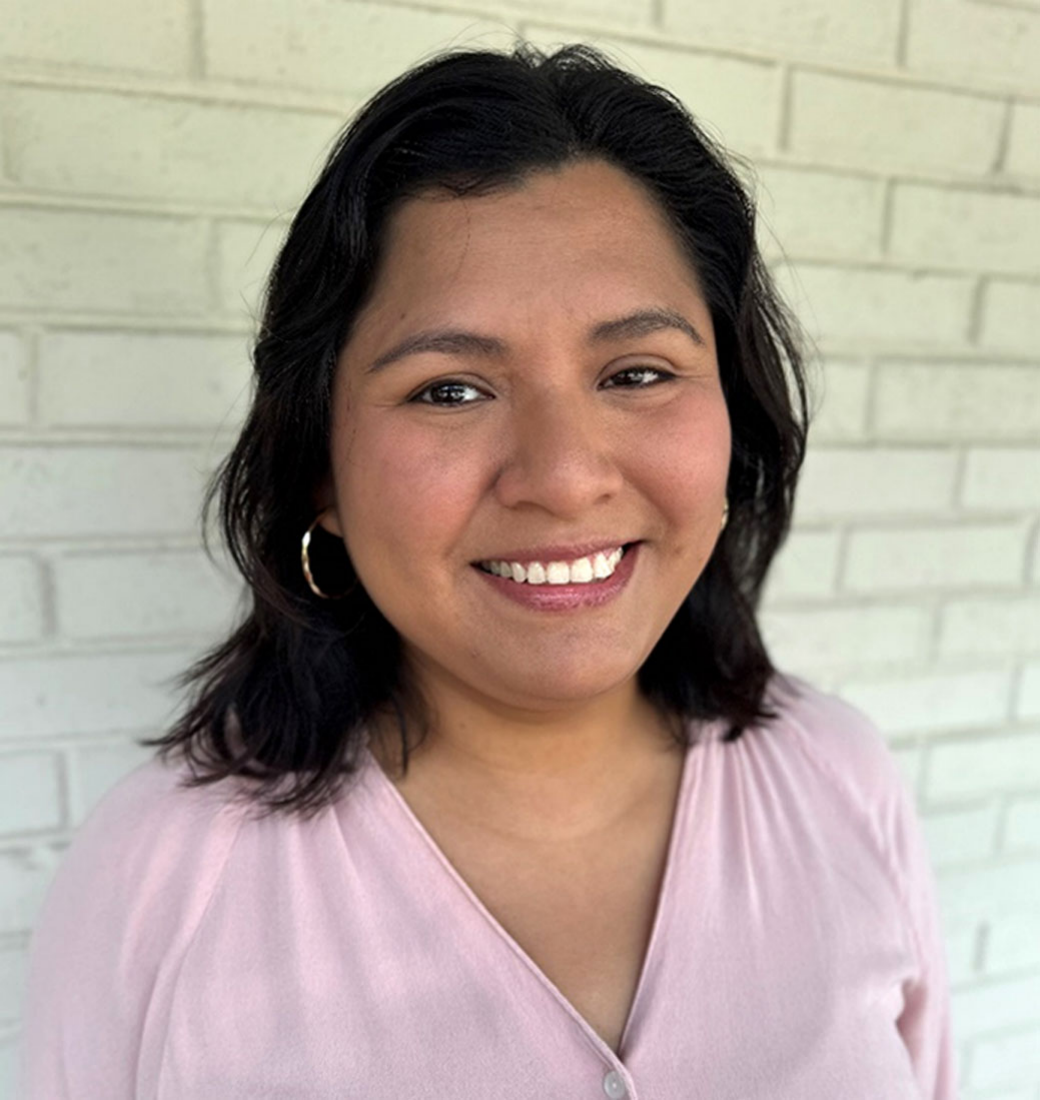


**Karen Ayala-Baylon**

Karen's scientific journey started at Homewood High School, in Alabama (AL, USA). Having immigrated to the United States in 2005, she sought comfort in STEM related courses and solidified her love of science. Thanks to her fun and passionate chemistry teacher, Karen decided to pursue a degree in this field and got accepted to the University of West Alabama (UWA), AL, USA. There, she gained research experience and developed various bench skills while working with Yun Ho Kim in the Chemistry Department, learning how to extract, purify and crystallize the components from the toothache tree (*Zanthoxylum clava-herculis*, a locally sourced tree known for its anaesthetic properties). Although Karen enjoyed this experience, she wanted to explore other areas of science glimpsed at during her Medical Sciences minor and realized her journey was not over.

After completing her B.S. in Chemistry at UWA, Karen searched for biology research opportunities and started volunteering at Lucas Pozzo-Miller's laboratory in the Department of Neurobiology at UAB, where she was later hired as a lab technician. This allowed her to acquire mouse husbandry skills, learn other techniques used in the lab, and gain insight into RTT research and disease models. Wanting to expand her career, Karen decided to pursue a Biology master's degree with Pozzo-Miller, investigating whether social memory deficits and atypical social behaviours seen in male *Mecp2*-knockout mice – used as an animal model for RTT – are also present in female *Mecp2* heterozygous (HET) mice. Furthermore, Karen explored whether the BDNF mimetic LM22A-4 can provide therapeutic improvements within the HET females. These results from her thesis were used for the research article published in DMM (Medeiros et al., 2024), now awarded with the 2024 Outstanding Paper Prize.

Following completion of her master's degree, Karen moved to North Carolina to pursue more translational approaches centred on human research. In 2022, she started working as a Clinical Studies Coordinator at Wake Forest Baptist Health (now Atrium Health Wake Forest Baptist), in the Department of Infectious Diseases. Here, she leads multiple Phase 1−2 clinical trials and ensures proper compliance with various regulatory entities. This work has given Karen a better understanding and appreciation for Translational Research, having experienced different stages within the clinical trial process. Although Karen's scientific journey detoured away from the bench, one thing remaining constant and unchanging is her love for science and research. With her journey still in progress, Karen hopes to continue contributing to the body of scientific knowledge for many more years with the findings from these trials.

### Hailey Egido-Betancourt


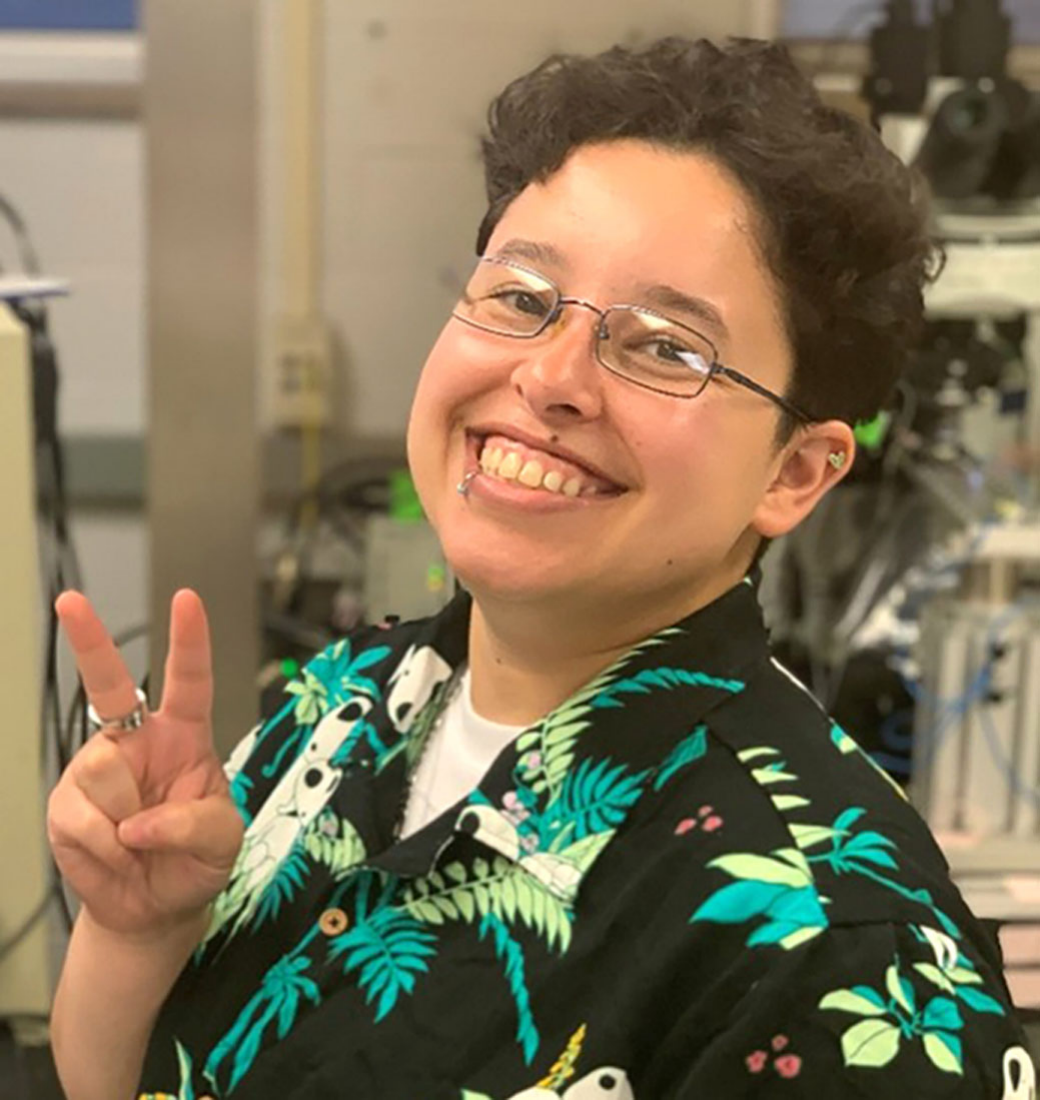


**Hailey Egido-Betancourt**

Hailey began their scientific endeavours while studying a bachelor's (B.S.) in biochemistry at the University of West Florida (UWF), FL, USA, in 2011. During this time, Hailey worked with Karen Molek (Department of Chemistry) on the synthesis and characterization of surface-modified zinc oxide quantum dots and, later, with Leonard W. ter Haar (Department of Chemistry) and Christopher Varney (Department of Physics), utilizing Monte Carlo and exact diagonalization methods in Cu^2+^ frustrated trimer spin systems. While being immersed in these projects, Hailey developed an interest in how different signalling pathways modulate neuronal activity, which led to an undergraduate internship at the University of Colorado Boulder (CO, USA) within the Summer Multicultural Access to Research Training (SMART) program, under Randall O'Reilly. This summer internship allowed Hailey to be immersed in the field of neuroscience, by learning how to align structural images to their fMRI counterparts and observing captured dopamine signals in the lateral habenula. By the end of the SMART program, Hailey became interested in seizures and autism spectrum disorders, and realized another opportunity was needed for growth within the field.

After completing the B.S. at UWF, in 2016, Hailey was accepted to the National Institute of General Medical Sciences Post-baccalaureate Research Education Program (NIGMS-PREP) at UAB. This program supports and enhances educational STEM activities in underrepresented backgrounds. Thus, Hailey joined the Neurobiology laboratory of Lucas Pozzo-Miller and worked on a project aimed at characterizing the mosaicism present within a rodent model of RTT, a neurodevelopmental disorder characterized by the loss of acquired language and motor abilities. Additionally, these individuals experience seizures and developmental delays in cognition, which continued to captivate Hailey's interests. Within the lab, they garnered skills with murine animals, immunohistochemistry and confocal microscopy, which contributed substantially to the findings in the DMM paper by Medeiros et al. (2024) winning the Outstanding Paper Prize. Along the way, Hailey became curious about the molecular mechanisms that can precede a seizure as well as mechanisms that manifest learning disabilities. Thus, they aimed to continue studying how seizures develop and pursued a Ph.D.

In 2017, Hailey got accepted into Wake Forest University's Neuroscience Graduate School program. There, they studied under Kimberly Raab-Graham (Department of Translational Neuroscience), investigating how Ca^2+^ affects branch variability in neurons afflicted with tuberous sclerosis complex (TSC). People with TSC experience a variety of issues, such as epilepsy and autism spectrum disorders, all of which are interests of Hailey's. This project, along with other side graduate projects, was exactly what Hailey needed to continue as a budding scientist within academia. Here, Hailey garnered a myriad of skills, such as stereotaxic surgery, Ca^2+^ imaging, electrophysiology and generating manuscripts, which facilitated acquiring a PhD and accepting a postdoctoral position.

Hailey is now a postdoctoral researcher at North Carolina Agricultural and Technical State University (NCAT), NC, USA under Farr Niere (Department of Biology). As part of the Niere lab, Hailey is studying the implications of an RNA binding protein, DJ1 and its downstream targets, in a preclinical model of TSC. Hailey is now learning how to conduct anxiety behavioural studies and assessing whether an anxiety phenotype is present in a mouse model with TSC. Hailey hopes to partner with other researchers at NCAT, bridge projects and continue growing their network.

### Christopher Chapleau


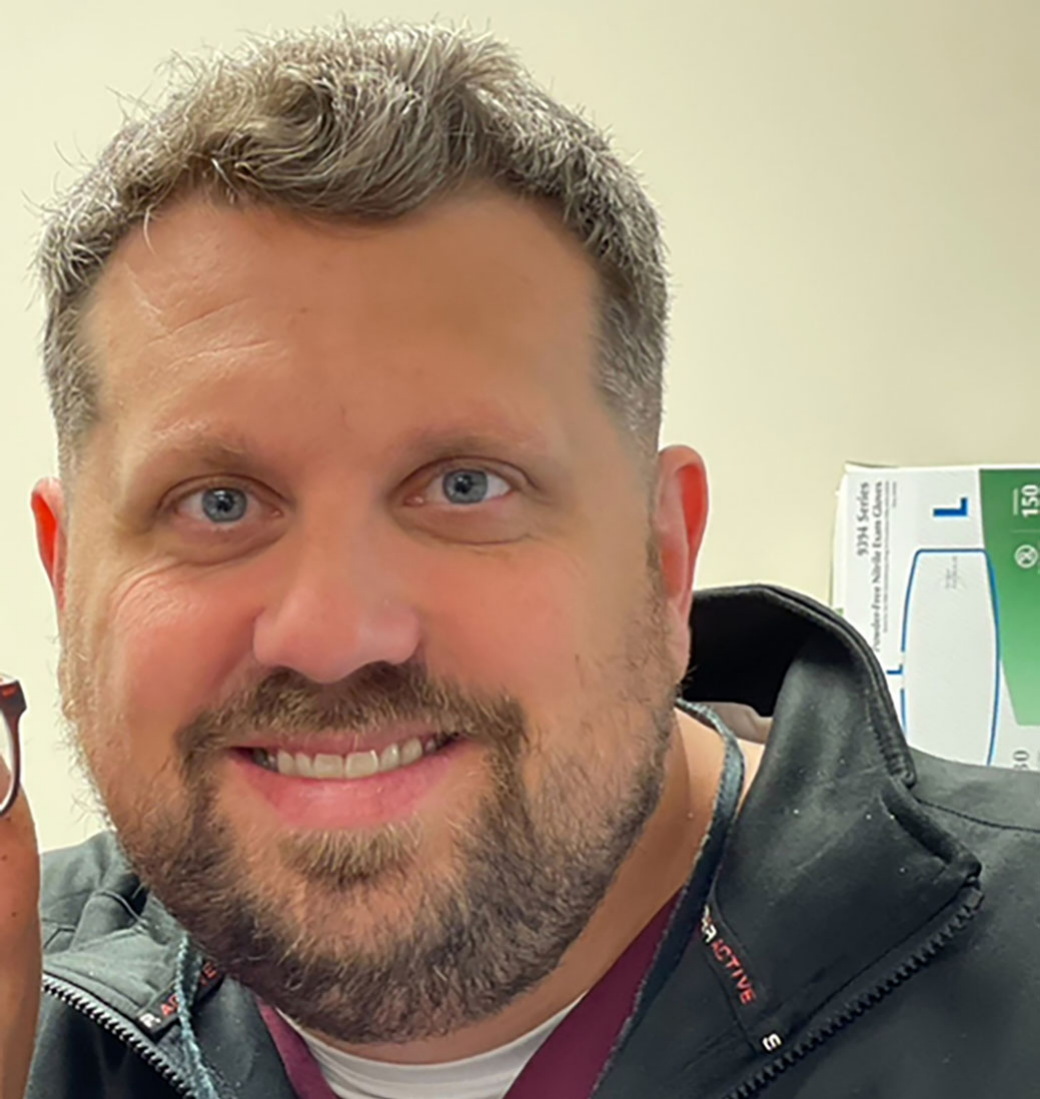


**Christopher Chapleau**

Chris is currently the Pharmacy Manager of the Division of Investigational Drug Services in the Department of Pharmacy at UAB. Prior to graduating from Samford University, McWhorter School of Pharmacy (AL, USA), Chris completed undergraduate studies in neuroscience, biology and psychology from Carthage College (WI, USA) and earned a graduate degree from UAB (PhD in 2008) under the mentorship of Lucas Pozzo-Miller. While in pharmacy school, Chris worked as a postdoctoral research fellow at the UAB under the mentorship of Lucas Pozzo-Miller and Alan Percy, focusing on the pathology and treatment of RTT. After graduating from pharmacy school, Chris completed a residency in pharmacy practice at UAB. Chris’ professional interests include the involvement of pharmacists in clinical trials and promoting patient safety during clinical trials.

### Wei Li


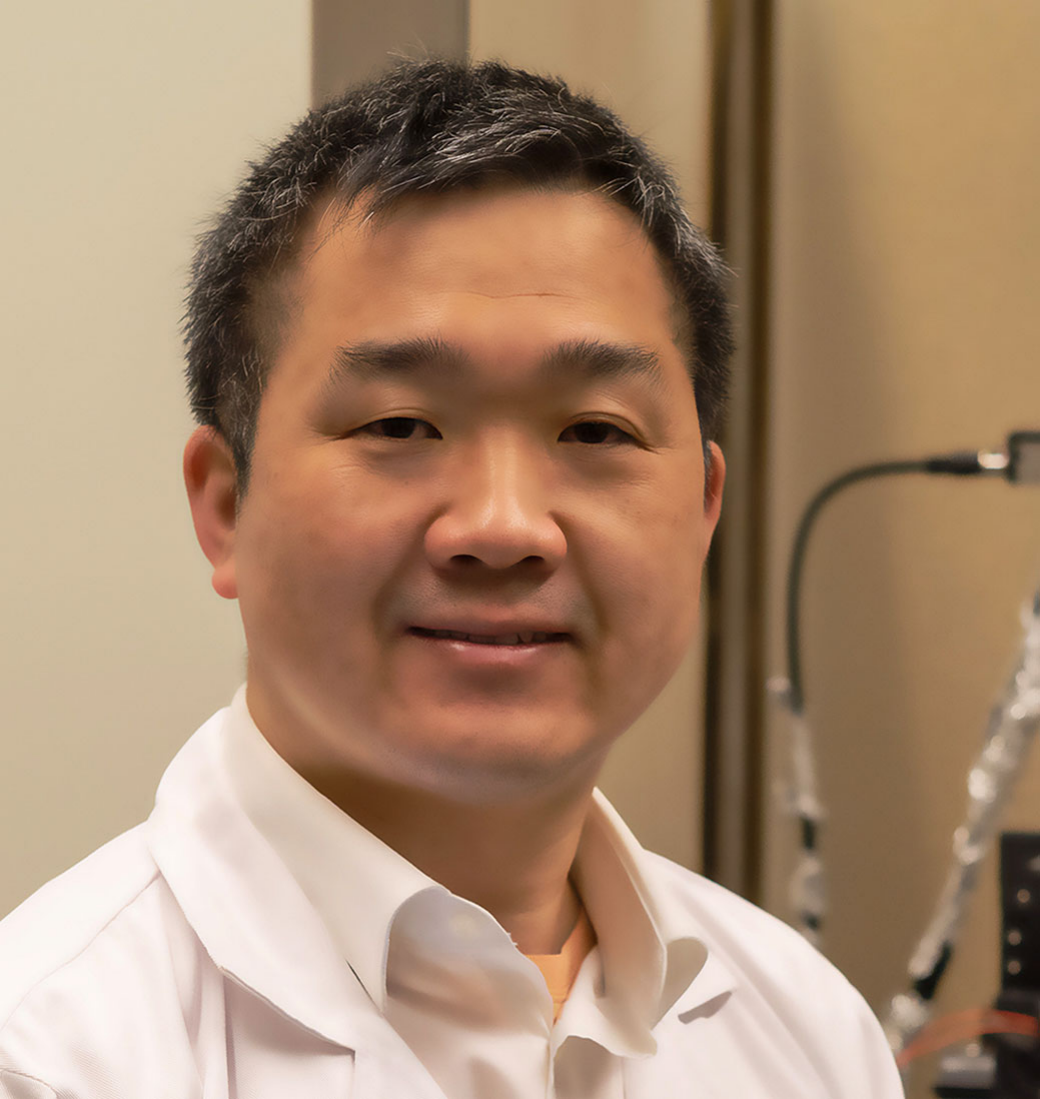


**Wei Li**

Wei is an Assistant Professor of Neurobiology at UAB, with a long-standing research interest in the molecular, cellular and circuit-level mechanisms that underlie brain function and dysfunction. His work focusses on the complex interplay between neurons and glia – particularly astrocytes – in neurodevelopmental disorders, such as RTT. He began his academic journey in China, where he earned a master's degree in Pharmacology from Zhejiang University (China). Wei later pursued a PhD in neuroscience at the University of South Dakota (SD, USA), where he investigated activity-dependent plasticity and BDNF-mediated α-amino-3-hydroxy-5-methyl-4-isoxazolepropionic acid receptor (AMPAR) trafficking, laying a strong foundation for his interest in synaptic regulation. Following his doctoral training, Wei joined Lucas Pozzo-Miller's lab at UAB as a postdoc, where he studied the pathophysiology of MECP2 mutations in mouse models of RTT. His work uncovered critical disruptions in BDNF signalling, excitation−inhibition balance and synaptic plasticity, thereby contributing to our understanding of the neurobiological basis of autism spectrum disorders.

As an independent investigator, Wei has expanded his research to explore the cerebellum's underappreciated role in social and motor deficits associated with RTT. His lab employs an interdisciplinary toolkit – including *in vivo* electrophysiology, optogenetics and behavioural analysis – to investigate how astrocyte−neuron signalling influences neural circuitry and behaviour. Recent publications from his group highlight novel findings on the regulation of Purkinje cell function by cerebellar astrocytes and the modulatory role of dopaminergic input on glial physiology. Wei's research has been consistently supported by the National Institutes of Health and foundations, such as the International Rett Syndrome Foundation. Beyond the bench, he is deeply committed to education and mentorship. He teaches in UAB's graduate neuroscience programs, and has mentored numerous undergraduate, graduate and post-baccalaureate students – many of whom have received institutional research awards. He also serves on editorial boards for several neuroscience journals. As a scientist, Wei continues to advance our understanding of glial contributions to neural function and aims to translate these insights into therapeutic strategies for neurodevelopmental disorders.

## Outstanding Paper Prize winner for Resources & Methods articles: Jasmin Scheurer and Birgit Sauer

### Jasmin Scheurer


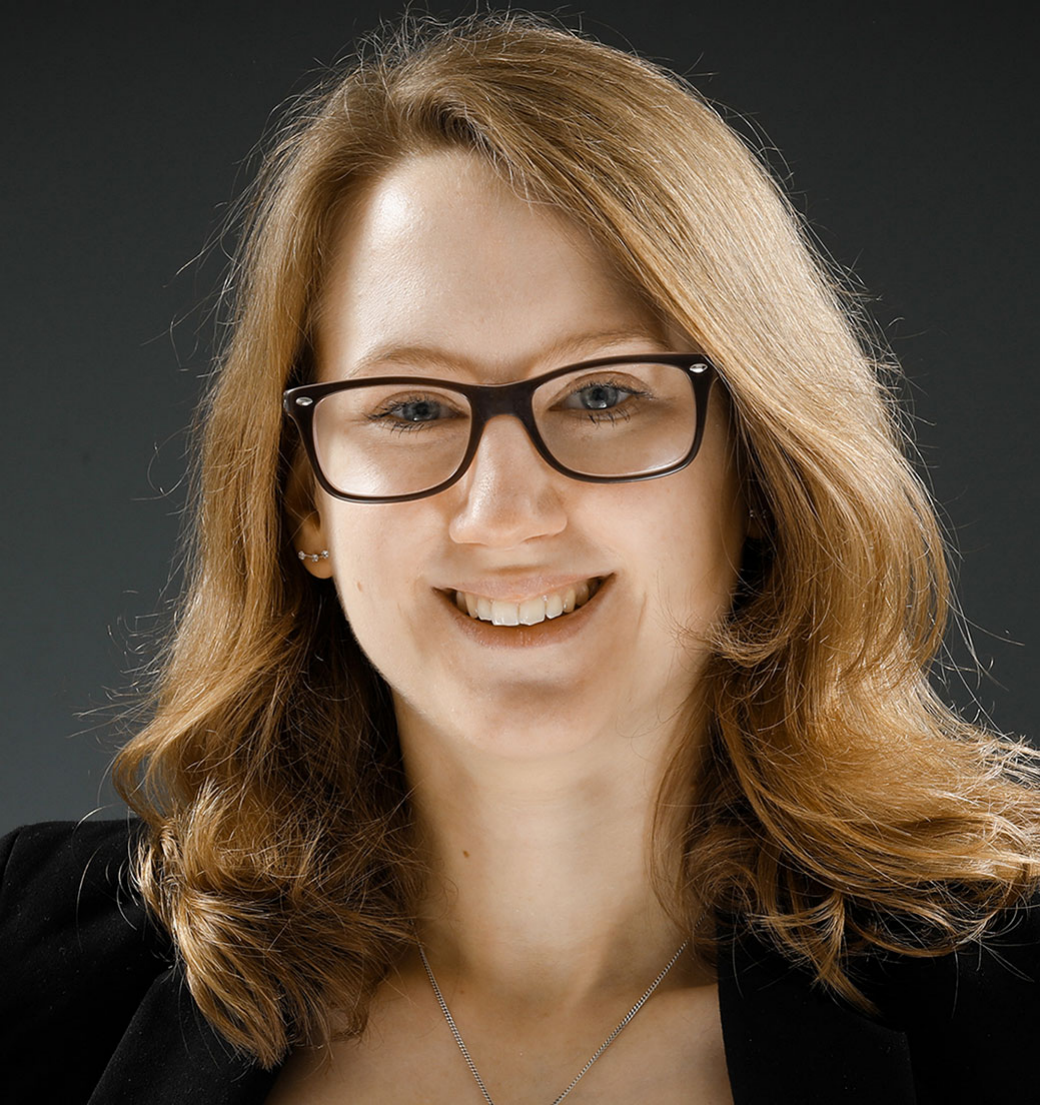


**Jasmin Scheurer**

Jasmin started her scientific journey in 2011, when she moved to Ulm, Germany and started studying molecular medicine, completing with a Master's degree. It was during this time, when she became particularly interested in the field of immunology. Jasmin's interest in immunology continued into her doctorate. From 2016−2020, she was associated to the Department of Paediatrics and Adolescent Medicine at Ulm University Medical Centre for her doctoral thesis and attended the International Graduate School for Molecular Medicine at Ulm University. During this time, she began to focus on the field of T-cell-induced graft-versus-host disease (GVHD) – a rare disorder that can affect immune-deficient or immune-suppressed people after a bone marrow transplant or non-irradiated blood transfusion – and analysed the therapeutic potential and mode of action of myeloid-derived suppressor cells and rapamycin in the prophylaxis of GVHD. Jasmin acquired knowledge in numerous immunological (e.g. flow cytometry) and cell biological methods (e.g. proliferation and apoptosis assays, cytokine and chemokine assays), as well as in animal models to investigate the pathogenesis and potential therapies.

In October 2020, Jasmin joined the laboratory team of Prof. Birgit Schittek (Department of Dermatology, University Hospital, Tübingen, Germany), for two years with her practical experience in immunology and T-cell-derived inflammatory diseases. The scientific focus of the project was on the development of inflammatory and immunocompetent human three-dimensional (3D) skin equivalents for laboratory studies of inflammatory skin diseases. Mouse and human skin differ significantly in cellular architecture and physiology, which makes it difficult to extrapolate research results obtained from mouse studies to humans. *In vitro* 3D human skin equivalents, including defined immune cell subsets, can bridge the gap between traditional 2D cell culture and animal models, and are more likely to reflect the complexity involved in human skin homeostasis.

This project was part of the Transregional Collaborative Research Centre ‘Transregio 156’ funded by the Deutsche Forschungsgemeinschaft (DFG). Researchers in this Transregional Collaborative Research Centre study how immune cells of the skin interact with each other as well as with other types of skin cell and the cutaneous microbiome. Their work is also aimed at providing new insights into how different types of skin cell influence other immune cells and, hence, how the body's multi-layered immune response functions in detail. The goal is to develop novel therapies to treat chronic inflammatory diseases, such as psoriasis or atopic dermatitis. As most of the projects within ‘Transregio 156’ try to translate their experimental data generated *in vitro* and by using mice into the human system, and because human tissue is only available in limited amounts, this leads to a significant demand for suitable human tissue models.

The work of Jasmin and Birgit Schittek's laboratory team within ‘Transregio 156’ gave rise to a Resources & Methods article published in DMM (Scheurer et al., 2024) that has now been awarded the Outstanding Paper Prize. In this work, the team advanced 3D human skin equivalents to model the inflammatory skin diseases atopic dermatitis and psoriasis by using cytokine stimulation, successfully integrating TH1 T-cells into skin models to develop an immunocompetent 3D psoriasis model. They performed in-depth histological and functional characterization of 3D skin equivalents and validated them in terms of tissue architecture, pathological changes, expression of antimicrobial peptides and colonization of *Staphylococcus aureus*, using 3D reconstruction by multiphoton microscopy and phenotyping by highly multiplexed co-detection by indexing (CODEX) microscopy. The team was able to show that their skin equivalents have a structural architecture with a well-developed dermis and epidermis, thus, resembling human skin. In addition, the skin models of atopic dermatitis and psoriasis show several phenotypic features of inflammatory skin disease, including disturbed epidermal differentiation and alterations in the expression of epidermal barrier genes and antimicrobial peptides, and can be reliably used to test novel treatment strategies.

### Birgit Sauer


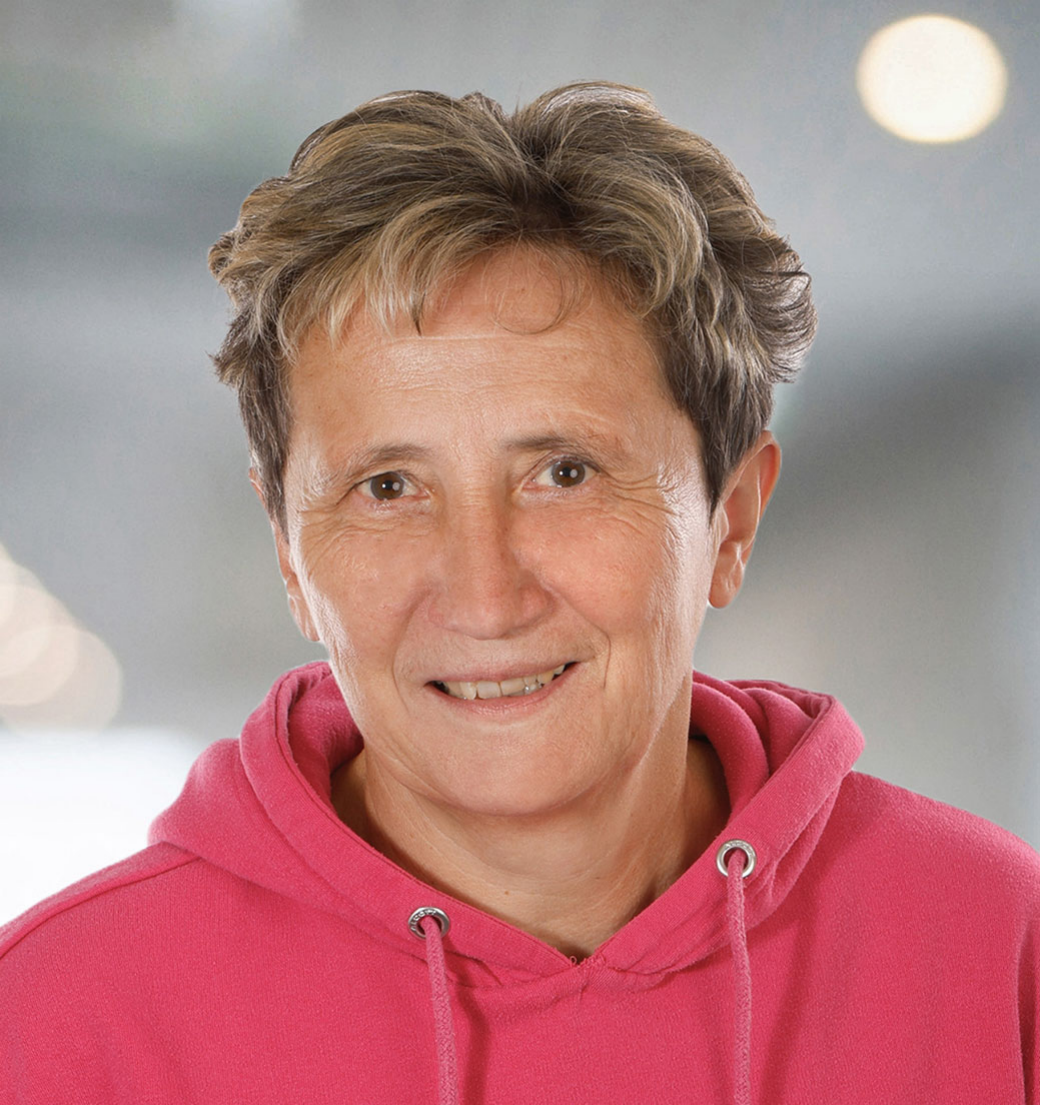


**Birgit Sauer**

Birgit finished her education as a biological technical research assistant in 1986 in at the Berufskolleg Olsberg für Technische Assistenten Olsberg, Germany. Her first job was at the Max-Planck Institute for Biological Cybernetics, Tübingen in the lab of Prof. Karl Götz. From November 1986 until September1988 she worked there for various people. She did behavioural studies on *Calliphora* and *Drosophila* including neuroanatomy and neuropharmacology investigations, gaining deeper insights into histology and electron microscopy.

This was followed by working 2.5 years at the University of California, Los Angeles (UCLA), CA, USA, investigating the auditory system of frogs (*Eleutherodactylus coqui* and *Rana pipiens*) in Prof. Peter Narins’ lab and mastering symbiotic nitrogen fixation of alfalfa and peas in Prof. Ann Hirsch's lab. During this time, she worked on the physiology of hearing using a frog model, including electrophysiological analyses of auditory nerve stimulation. She accomplished lots of different molecular biology techniques, such as PCR, which were right in the beginning of their development. While at UCLA she published her first own paper (Bochenek and Hirsch, 1990).

In January 1991, she moved back to Germany and started in the lab of Prof. Christiane Nüsslein Volhard (Max-Planck-Institut für Entwicklungsbiologie (since 2021 Max-Planck-Institut for Biologie Tübingen), on the mutagenesis of zebrafish (*Brachydanio rerio*), where she successfully isolated mutants with defects in the early embryonic development. After three years, in 1994, she changed to the lab of Prof. Axel Borst (then at the Friedrich Miescher Laboratory, Max Planck Society, Tübingen) and collaborated with several people researching the visual system of *Calliphora erythrocephala*.

In 1996, Birgit joined the group of Prof. Claus Garbe/Prof. Birgit Schittek at the University of Tübingen, Department of Dermatology. Since then, she has worked in Birgit Schittek's group, participating in several projects investigating the molecular and cellular mechanisms of progression and therapy resistance of melanoma, and innate immunity of the skin. She is co-author on several papers, including (Schittek et al., 2001; Bitschar et al., 2019). Since 2018, she has been working together with Jasmin Scheurer, in Birgit Schittek's group, on human 3D skin models in a service project as part of the TR156 funded by the DFG. They advanced their existing 3D models to model inflammatory skin diseases including fibrosis, atopic dermatitis and psoriasis and were successful in the integration of immune cells such as T cells, macrophages or Langerhans cells into 3D human skin models. Using these advanced 3D skin models, they were able to simulate several human inflammatory skin diseases in a physiological environment.

**DMM Prize 2024 shortlist**

**Research Articles**

**A small-molecule TrkB ligand improves dendritic spine phenotypes and atypical behaviors in female Rett syndrome mice.**

Destynie Medeiros, Karen Ayala-Baylon, Hailey Egido-Betancourt, Eric Miller, Christopher Chapleau, Holly Robinson, Mary L. Phillips, Tao Yang, Frank M. Longo, Wei Li and Lucas Pozzo-Miller. *Dis. Model Mech.* (2024) 17, dmm050612. doi:10.1242/dmm.050612

**Characterization of a monkey model with experimental retinal damage induced by N-methyl-D-aspartate.**

Guo Liu, Longxiang Huang, Junkai Tan, Yun Wang, Chunlin Lan, Yaxi Chen, Yukai Mao, Xizhen Wang, Ning Fan, Yihua Zhu, Xianjun Zhu and Xuyang Liu. *Dis. Model Mech.* (2024) 17, dmm050033. doi:10.1242/dmm.050033

**Distinct fingerprints of tRNA-derived small non-coding RNA in animal models of neurodegeneration.**

Sharada Baindoor, Hesham A. Y. Gibriel, Morten T. Venø, Junyi Su, Elena Perez Morrissey, Elisabeth Jirström, Ina Woods, Aidan Kenny, Mariana Alves, Luise Halang, Paola Fabbrizio, Maria Bilen, Tobias Engel, Marion C. Hogg, Caterina Bendotti, Giovanni Nardo, Ruth S. Slack, Jørgen Kjems and Jochen H. M. Prehn. *Dis. Model Mech.* (2024) 17, dmm050870. doi:10.1242/dmm.050870

**The role of mesenchymal cells in cholangiocarcinoma.**

Mireia Sueca-Comes, Elena Cristina Rusu, Jennifer C. Ashworth, Pamela Collier, Catherine Probert, Alison Ritchie, Marian Meakin, Nigel P. Mongan, Isioma U. Egbuniwe, Jesper Bøje Andersen, David O. Bates and Anna M. Grabowska. *Dis. Model Mech.* (2024) 17, dmm050716. doi:10.1242/dmm.050716

**A genetically small fetus impairs placental adaptations near term.**

Ionel Sandovici, Olatejumoye Knee, Jorge Lopez-Tello, Norman Shreeve, Abigail L. Fowden, Amanda N. Sferruzzi-Perri and Miguel Constância. *Dis. Model Mech.* (2024) 17, dmm050719. doi:10.1242/dmm.050719

**A deleterious variant of *INTS1* leads to disrupted sleep–wake cycles.**

Shir Confino, Yair Wexler, Adar Medvetzky, Yotam Elazary, Zohar Ben-Moshe, Joel Reiter, Talya Dor, Simon Edvardson, Gali Prag, Tamar Harel and Yoav Gothilf. *Dis. Model Mech.* (2024) 17, dmm050746. doi:10.1242/dmm.050746

**Reduced connexin-43 expression, slow conduction and repolarisation dispersion in a model of hypertrophic cardiomyopathy.**

Seakcheng Lim, Melissa M. Mangala, Mira Holliday, Henrietta Cserne Szappanos, Samantha Barratt-Ross, Serena Li, Jordan Thorpe, Whitney Liang, Ginell N. Ranpura, Jamie I. Vandenberg, Christopher Semsarian, Adam P. Hill and Livia C. Hool. *Dis. Model Mech.* (2024) 17, dmm050407. doi:10.1242/dmm.050407

***Smad4* restricts injury-provoked biliary proliferation and carcinogenesis.**

William B. Alexander, Wenjia Wang, Margaret A. Hill, Michael R. O'Dell, Luis I. Ruffolo, Bing Guo, Katherine M. Jackson, Nicholas Ullman, Scott C. Friedland, Matthew N. McCall, Ankit Patel, Nathania Figueroa-Guilliani, Mary Georger, Brian A. Belt, Christa L. Whitney-Miller, David C. Linehan, Patrick J. Murphy and Aram F. Hezel. *Dis. Model Mech.* (2024) 17, dmm050358. doi:10.1242/dmm.050358

**Generation of a zebrafish neurofibromatosis model via inducible knockout of *nf2a/b*.**

Ayyappa Raja Desingu Rajan, Yuanyun Huang, Jan Stundl, Katelyn Chu, Anushka Irodi, Zihan Yang, Brian E. Applegate and Marianne E. Bronner. *Dis. Model Mech.* (2024) 17, dmm050862. doi:10.1242/dmm.050862

***Staphylococcus aureus* lipid factors modulate melanoma clustering and invasion in zebrafish.**

Morgan A. Giese, Gayathri Ramakrishnan, Laura H. Steenberge, Jerome X. Dovan, John-Demian Sauer and Anna Huttenlocher. *Dis. Model Mech.* (2024) 17, dmm050770. doi:10.1242/dmm.050770

**Resources & Methods articles**

**A prioritization tool for cilia-associated genes and their *in vivo* resources unveils new avenues for ciliopathy research.**

Robert E. Van Sciver and Tamara Caspary. *Dis. Model Mech.* (2024) 17, dmm052000. doi:10.1242/dmm.052000

**Establishing mouse and human oral esophageal organoids to investigate the tumor immune response.**

Yuan Jiang, Hua Zhao, Shuai Kong, Dan Zhou, Jinxiu Dong, Yulan Cheng, Shuo Zhang, Fei Wang, Andrew Kalra, Nina Yang, Dan-Dan Wei, Jian Chen, Yuan-Wei Zhang, De-Chen Lin, Stephen J. Meltzer and Yan-Yi Jiang. *Dis. Model Mech.* (2024) 17, dmm050319. doi:10.1242/dmm.050319

**Histological and functional characterization of 3D human skin models mimicking the inflammatory skin diseases psoriasis and atopic dermatitis.**

Jasmin Scheurer, Birgit Sauer, Jule Focken, Martina Giampetraglia, Annika Jäger, Christian M. Schürch, Bettina Weigelin and Birgit Schittek. *Dis. Model Mech.* (2024) 17, dmm050541. doi:10.1242/dmm.050541

**Winner: Research Article**

**A small-molecule TrkB ligand improves dendritic spine phenotypes and atypical behaviors in female Rett syndrome mice.**

Destynie Medeiros, Karen Ayala-Baylon, Hailey Egido-Betancourt, Eric Miller, Christopher Chapleau, Holly Robinson, Mary L. Phillips, Tao Yang, Frank M. Longo, Wei Li and Lucas Pozzo-Miller. *Dis. Model Mech.* (2024) 17, dmm050612. doi:10.1242/dmm.050612

**Winner: Resources & Methods article**

**Histological and functional characterization of 3D human skin models mimicking the inflammatory skin diseases psoriasis and atopic dermatitis.**

Jasmin Scheurer, Birgit Sauer, Jule Focken, Martina Giampetraglia, Annika Jäger, Christian M. Schürch, Bettina Weigelin and Birgit Schittek. *Dis. Model Mech.* (2024) 17, dmm050541. doi:10.1242/dmm.050541
